# Implementation of a Customized Safety Checklist in Gastrointestinal Endoscopy and the Importance of Team Time Out—A Dual-Center Pilot Study

**DOI:** 10.3390/medicina59061160

**Published:** 2023-06-16

**Authors:** Irina Florina Cherciu Harbiyeli, Daniela Elena Burtea, Mircea-Sebastian Serbanescu, Carmen Daniela Nicolau, Adrian Saftoiu

**Affiliations:** 1 Research Centre of Gastroenterology and Hepatology, University of Medicine and Pharmacy of Craiova, 200638 Craiova, Romania; cherciuirina@gmail.com (I.F.C.H.); adriansaftoiu@gmail.com (A.S.); 2 Department of Medical Informatics and Biostatistics, University of Medicine and Pharmacy of Craiova, 200638 Craiova, Romania; mircea_serbanescu@yahoo.com; 3 Lotus Image Medical Center, Actamedica SRL, 540084 Targu Mures, Romania; carmen.nicolau@gmail.com

**Keywords:** GI endoscopy, safety checklist, team time out, patient safety

## Abstract

*Background and Objectives:* Checking and correctly preparing the patient for endoscopic procedures is a mandatory step for the safety and quality of the interventions. The aim of this paper is to emphasize the importance and necessity of a “team time out” as well as the implementation of a customized “checklist” before the actual procedure. *Material and Methods:* We developed and implemented a checklist for the safe conduct of endoscopies and for the entire team to thoroughly know about the patient’s medical history. The subjects of this study were 15 physicians and 8 endoscopy nurses who performed overall 572 consecutive GI endoscopic procedures during the study period. *Results:* This is a prospective pilot study performed in the endoscopy unit of two tertiary referral medical centers. We customized a safety checklist that includes the steps to be followed before, during and after the examination. It brings together the whole team participating in the procedure in order to check the key points during the following three vital phases: before the patient falls asleep, before the endoscope is inserted and before the team leaves the examination room. The perception of team communication and teamwork was improved after the introduction of the checklist. The checklist completion rates, identity verification rates of patients by the endoscopist, adequate histological labeling management and explicit recording of follow-up recommendations are some of the parameters that improved post-intervention. *Conclusions:* Using a checklist and adapting it to local conditions is a high-level recommendation of the Romanian Ministry of Health. In a medical world where safety and quality are essential, a checklist could prevent medical errors, and team time out can ensure high-quality endoscopy, enhance teamwork and offer patients confidence in the medical team.

## 1. Introduction

Endoscopic procedures represent an important part of daily practice for both gastroenterologists and nurses, enabling the timely diagnosis and precise treatment of digestive diseases. In the past 20 years, the possibilities of diagnosing and treating digestive diseases have increased considerably; GI (gastro-intestinal) endoscopy procedures have become more complicated and varied, hence, the concerns about the quality of medical care and patient safety have increased [1]. In recent years, there has been a growing interest in the possibilities of preventing errors in diagnostic and interventional endoscopy suites. The knowledge about and awareness of patient safety risk factors are essential in order to improve and enhance the GI endoscopy team, the working environment and, finally, the endoscopic performance.

The diversity of digestive pathology as well as the fast growth of the therapeutic endoscopic opportunities led to more evolved and complex procedures. The practical challenges of advanced endoscopy are approaching the complexity of a surgery. An advanced endoscopy is similar to surgery in terms of the technical complexity and invasiveness. This contains a higher risk of adverse events associated with the therapeutic interventions [2]. The patients count on the endoscopy team to execute the GI endoscopy procedures in a secure, rigorous and standardized manner. Given the growing number of GI endoscopic procedures, many of which are technically complex, as well as the aging patient population and its increased prevalence of comorbidities, the GI endoscopy safety checklists have become more popular in recent years. Regardless of the wide acknowledgement of the significance of GI endoscopy safety checklists, there is little information regarding the actual implementation among the GI endoscopy units [3,4,5].

A modern endoscopy unit needs extremely competent leaders, supported by excellent teams who can handle a high volume of procedures safely, efficiently and for the benefit of the patient. In gastroenterology, quality and safety are crucial issues that are motivated by a shared desire to advance best practices and make it easier for patients to receive evidence-based care. Patient safety should not be compromised in light of the development of endoscopic practice, and efforts to uphold and enhance GI endoscopy safety should always be made [6,7]. Additionally, it is very important that the team understand and accept that some of the endoscopic procedures, especially advanced GI endoscopic procedures, have a higher risk of adverse events compared to regular endoscopies [1,8].

The advanced endoscopy team is part of a bigger service that provides access to therapeutic intervention, and the staff should already have experience, planned strategies and skills to deal with potential adverse events or failed procedures. If the adverse events result from inadequate planning of the intervention rather than a lack of technical skills, quality improvement initiatives may help to reduce such complications [9,10].

The results are determined not only by the skills of the endoscopist, but by the effectiveness of the team.

An effective endoscopy service depends on the following [7]:

- The endoscopy team;
- Team leaders;
- The institution where the service operates;
- The political context.

The team itself consists of nurses, technical staff, administrative staff, anesthesiologists and endoscopic physicians. Some of the endoscopists may spend half a day or one day per week in the endoscopy department. The temporary nature of some team members can affect the team dynamics, leading to an increased disruption in the workplace. As a result, the team may not function efficiently. Providing a modern endoscopy service requires effective leadership and teamwork [11,12].

Each endoscopy unit has the possibility to draw up its own checklist, either in a printed or electronic version. Most checklists in digestive endoscopy do not specifically address advanced procedures; therefore, they need to be nuanced/individualized [13,14]. According to the World Health Organization, the implementation of customized safety checklists can improve procedural outcomes and reduce human errors [15]. Several studies state that the implementation of a GI endoscopy safety checklist significantly improved endoscopy team communication and teamwork. It is possible to extrapolate that with improved team communication, medical errors may be reduced, thereby preventing adverse events [2,14]. However, there is no guidance on how best to implement GI endoscopy checklists or solid high-level data regarding any measure of their usefulness including mortality rates, adverse event rates or endoscopy completion rates. Hence, there is a real need for more studies centered on assessing not only the usefulness of an endoscopy safety checklist, but also for identifying the best implementation strategies.

In this paper, we aim to formally evaluate the feasibility of successfully implementing a customized safety checklist in our dual center GI endoscopy units, to identify strategies and address barriers for facilitating checklist compliance and to summarize the impact on team communication and commitment to patients’ safety culture.

## 2. Material and Methods

This is a prospective pilot study performed in the endoscopy unit of two tertiary referral medical centers (Research Center of Gastroenterology and Hepatology, Craiova, Romania and Lotus Image Medical Center, Targu Mures, Romania).

This initiative was designed and approved as a quality improvement study, with the study subjects set to be the endoscopy staff comprising gastroenterology and anesthesia physicians and endoscopy nurses. The subjects from both endoscopy teams were enrolled at the same time in October 2021. The endoscopy staff members were included in the present study if they performed at least one endoscopy procedure and took part in each phase of the study. No patient information was collected during this study, hence, the exempt of approval from the Local Institutional Ethical Committee. The authors did not have access to information that could identify individual participants during or after data collection.

The case diversity included elective inpatients and outpatients who underwent diagnostic and therapeutic endoscopies. All procedures were performed with the patients under propofol sedation, administered by an anesthesiologist.

**Figure 1:**
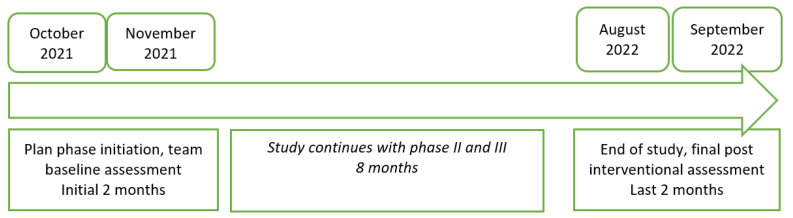
Study timeline.

Real-time observation of each endoscopic procedure was performed by one designated staff member, and data were collected from October 2021 to the end of September 2022, as described in Figure 1. The observations were performed on random consecutive days, with the aim of observing the widest range of physician–nurse combinations. There was no established scale to rate the difficulty of the endoscopy procedures. Rather, it was a personal evaluation made by each physician. The person responsible for completing the checklist asked the physicians “Do you consider the procedure difficult?’’ and they had the option to provide a Yes or No answer. The difficulty of the endoscopic procedure was assessed by each physician according to their knowledge and experience. It took into consideration factors such as poor bowel preparation; anatomical gastrointestinal alterations; the existence of tumors challenging to be traversed with the endoscope; polyps, which are hard to resect due to the size, number and location (characterized by using the SMSA classification); chronic alcoholic patients requiring higher sedation doses and breaks during the procedure due to the agitated status; etc.

The observational studies were prospectively conducted and reported following the STROBE statement. Initially, the baseline compliance rates were inspected during the first 2 months of the study. The final data were cross-checked by the multidisciplinary team established in both institutions, and the final assessment was performed during the last 2 months. Pre- and post-intervention staff compliance rates were compared. Team satisfaction and perception of the checklist was assessed by conducting a 5-question survey, after the quality improvement interventions were applied. The staff was asked if the checklist either caused time delay, improved patients’ safety, proved to be useful if they were to have an endoscopy or if, overall, they were satisfied with the checklist implementation process.

**Figure 2:**
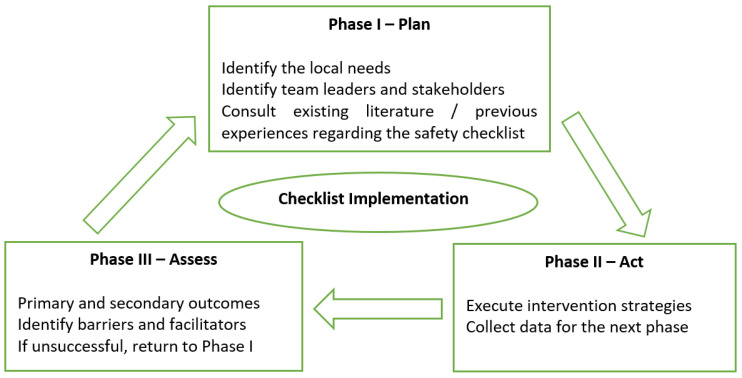
Study design.

The framework for the implementation of the safety checklist is described in Figure 2. Firstly, the scarce existing literature regarding the topic of this paper was consulted, and a personalized checklist was developed in order to be used in our two centers. After reaching an agreement regarding the final form of the checklist, we proceeded to the implementation stage, all with the goals to safely conduct endoscopies and for the entire team to thoroughly know the patient’s medical history.

Intervention strategies for the implementation of our prospectively developed checklist are as follows:

- Development and adjustment of the endoscopy checklist;
- Checklist introduction seminars;
- Team training sessions mandatory for all endoscopy staff;
- Physical reminders spread around the endoscopy department;
- Printed paper-based checklists available in a clear designated area;
- Advocating the use of a safety checklist as part of the endoscopy department policy;
- Mandating a single individual (physician or nurse) to lead the checklist process;
- Actively engaging the entire endoscopy team to contribute to all phases of the checklist.

Primary and secondary outcomes expected to be analyzed during the study included the following: (a) checklist completion rate, (b) identity verifications of patients, (c) validity of the endoscopy procedure appropriateness, (d) colonic preparation (Boston Bowel Preparation Scale—BBPS was used for the assessment of the colonic preparation), (e) colonoscopy completion rate and adenoma detection rate, (f) staff satisfaction and perception about the checklist, (g) peri-procedural complications (bleeding, perforation, cardiovascular events, death), (h) adequate histological labeling management, (i) explicit recording of follow-up recommendations (post-polypectomy surveillance, follow-up interval after colonoscopy with inadequate preparation, colorectal cancer screening, follow-up endoscopy for varices for patients with cirrhosis, follow-up after EUS for pancreatic lesions, etc.).

### Statistical Analysis

As all collected data were categorical, comparisons between groups (nurses versus physicians) were performed using chi-square test and, alternatively, Fisher’s exact test, when not all the parameters of the chi-square test were fulfilled. A *p*-value of <0.05 was considered to be significant.

For the staff questionnaires, the frequencies and proportions of responses were computed including only the response alternatives “Yes” and “No”, and ignoring the “Hesitant” responses.

Data collection was performed using Microsoft Office Excel 2019, (Microsoft Corp.; Redmond, WA, USA), while the statistical assessment was carried out using MATLAB 2021a (MathWorks Inc.; Natick, MA, USA).

## 3. Results

**Figure 3:**
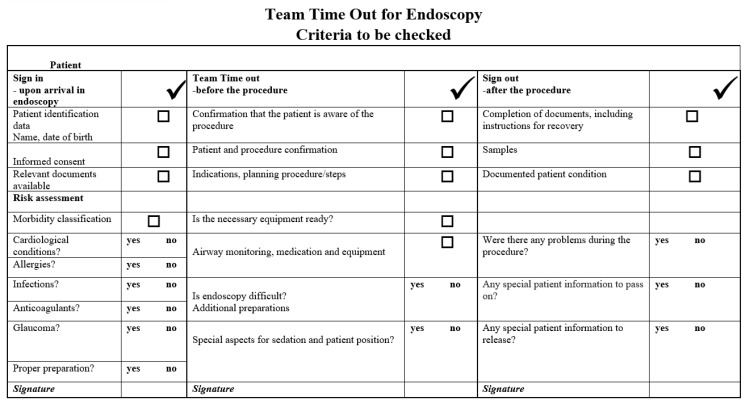
Our customized endoscopy checklist.

Based on the existing checklists published in the literature and the constant feedback received during the study, we developed a personalized checklist that includes the steps to be followed before, during and after the endoscopic examination (Figure 3). Although only one designated person was responsible to physically tick the required information and lead the process (either a physician or a nurse), the checklist drew together the whole team participating in the procedure in order to check the key points during the following three vital and equally important phases: before the patient falls asleep (‘’Sign in’’), before the endoscope is inserted (‘’Team out’’) and before the team leaves the examination room (‘’Sign out’’).

The “Sign in” phase starts once the patient enters the endoscopy room and comprises the following: (a) verification of patient’s identity, (b) completion of the informed consent forms, (c) verification of patient’s relevant medical documents, (d) morbidity classification according to the American Society of Anesthesiologists score, (e) assessment of relevant comorbidities (cardiopulmonary risks, presence of implantable medical devices, etc.), (f) any known allergies (including medication or previous difficulties with sedation/anesthesia), (g) usage of anticoagulant/antiplatelet medications, (h) a review of known infections (hepatitis, HIV, tuberculosis, COVID-19, etc.) and (i) confirmation of proper bowel preparation.

The “Time out” phase occurs before the endoscope is inserted and/or before the induction of sedation/general anesthesia. It includes the following: (a) confirmation that the patient is aware of the planned endoscopic procedure, (b) patient and procedure validation, (c) confirmation of indications, aims, potential limitations and summary of each step of the procedure, (d) confirmation that all required endoscopy equipment, endoscope(s) and accessories are available and properly functioning, (e) confirmation that the patient monitoring equipment is prepared and operational (functioning intravenous access, pulse oximetry, blood pressure, cardiac monitor), (f) inspection of any special aspects regarding sedation or patient position and (g) assessment of whether the endoscopic procedure is difficult and whether additional preparations are required.

The “Sign out” phase takes place in between the end of the endoscopic procedure and prior to the team exiting the endoscopy lab. This phase includes the following: (a) confirmation that the endoscopy report is accurate, including post-procedure instructions for patient’s recovery and eventual follow-up procedures that could be indicated, (b) confirmation that all histological samples collected during the endoscopy procedure are correctly labelled and documented, (c) documentation of patient’s post-procedure status and if any problems occurred during the procedure and (d) confirmation that all the necessary documents and information were passed on to the patient.

A mixed team dedicated to quality improvement reviewed both the local and already published data in order to identify the barriers and facilitators which might influence the checklist compliance (Table 1). In consequence, an implementation strategy was developed for overcoming the potential barriers. Within the strategy, we established a target for the checklist adherence rate (>75%). Supporting the team members who consistently used the checklist and debriefing the non-cooperative staff were important parts of achieving our target.

The study’s outcome rates were calculated after completing 572 consecutive endoscopic procedures, including 126 procedures during the baseline period and 446 procedures until the end of the study period. The assessment of the checklist implementation is presented in Table 2. There was a significant increase in the checklist completion rate (25% to 89%) after the intervention procedures. The identity verification of patients by the endoscopist also reached a significant increase (15% at baseline up to 90%) and remained high among nurses (98% at baseline to 99% at the end of the study). The baseline adenoma detection rate was 0.36, and the post-intervention rate was 0.39, hence, there was no significant statistical difference. The adequate histological labeling management and explicit recording of the follow-up recommendations are two parameters which had statistically significant improvements post-intervention, similar with the documentation of the colonic preparation and the assessment of the procedure validity.

**Figure 4:**
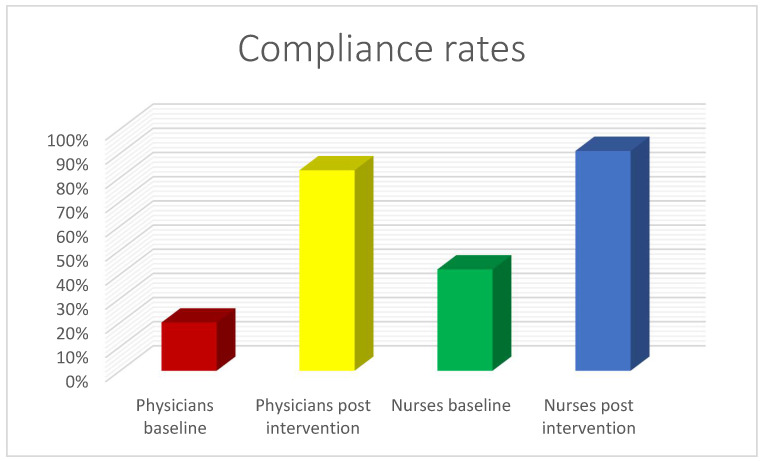
Compliance rates among physicians versus nurses.

The subjects of this study were 15 physicians (gastroenterologists and anesthesiologists) and 8 endoscopy nurses who worked at the endoscopy units in varying degrees of frequency and performed, overall, 572 consecutive GI endoscopic procedures during the study period. When assessing the compliance rates of the physicians versus the nurses, at baseline and post-intervention, an increase from 20% to 83% (*p* < 0.05) was noted among the physicians, and from 42% to 91% (*p* < 0.05) among nurses (Figure 4).

The survey conducted to assess the team satisfaction and perception of the checklist, after the quality improvement intervention, revealed that the perception of team communication and teamwork was improved after the checklist introduction; it denied the initial fears that the checklist might cause an extensive time delay, all the staff agreed that the checklist improves patient safety and the majority of the team would consider the checklist useful if they were to have an endoscopic procedure (Table 3).

## 4. Discussions

With increasing indications and more technically advanced gastrointestinal endoscopy techniques, finding strategies to prevent adverse events is an important aspect of the current clinical practice. In many places, the endoscopy has developed in the ad hoc way, following a variety of business models. Although an endoscopy team is a little unusual compared to other diagnostic health services, the team members still need to work in a coordinated and organized way, they must be trained to do their jobs, be motivated to excel, know what is expected from them and know their responsibilities [16].

Communication is important for both the safety and effectiveness of the GI endoscopic procedure. Studies on communication remain limited even in the field of surgery, where there is a much higher flow of complex procedures performed in teams [8,11,17]. An advanced endoscopic intervention would benefit from strong leadership and teamwork skills. One possible way to achieve this is for the endoscopist to adopt conscious verbal competence. An experienced endoscopist could defuse the team by starting a good flow of communication through a full team briefing even before the patient arrives, so that there is no distraction when the procedure begins [18,19].

The concept of verbal conscious competence may be relevant to the expert endoscopist, especially during the team time out process. The complexity of performing a polypectomy, for example, can be classified using the parameters of size, morphology, site and access (SMSA). Furthermore, SMSA classification can be used to estimate the time required to perform an advanced polypectomy along with assessing the difficulty of the procedure as it is mentioned in the Team Time Out phase of the endoscopy checklist. The following would be an illustrative example of performing an endoscopic mucosal resection: Before starting, the endoscopist describes their impression, whether they expect the polypectomy to be simple, moderately challenging or difficult. This first impression may not be definitive and may change during the treatment, at which point the endoscopist should provide an update to the team. The planned technical approach is described, including where the injection needle will be used for mucosal elevation, the intended effect and positioning of the polyp after elevation, the size of the polypectomy loop and the electrical settings for cutting and/or coagulation, etc. The endoscopist continues to discuss the likelihood of adverse events (e.g., bleeding, perforation, or incomplete resection), how these will be solved (e.g., adrenaline, clips, argon plasma, loop tip coagulation, additional cutting, surgical consult, etc.) and once again, asks for team confirmation that all the equipment is available and that the staff is competent and trained [20,21]. It is also recommended that at this point, the endoscopist encourages the team members to state whether they agree with the plan, to express their uncertainties and ask questions or to clearly articulate their opinions. Once a consensus of the whole team is reached and the start is confirmed, the endoscopist gives a verbal signal for the start of the therapy—“let us begin”. This is a practical example of how the Team Time Out phase should progress during the process of completing the endoscopy checklist.

Similar to the majority of previous published studies evaluating safety checklists in the GI endoscopy setting [22,23,24], our study did not find a clear association between the checklist implementation and the clinical outcomes. The staff members were worried that the patients would grow anxious if they were repeatedly asked a lot of questions, but this did not seem to be the case. This was just a secondary observation, considering that the patients were not the subjects of this study, although a questionnaire addressing the patients’ perspectives on the safety checklist would bring an added value to the study. As revealed in other published materials, in our study, the compliance rates of the nurses were higher than the physician compliance rates [12,22], but we noticed an increase in the identity checks performed by the physicians, which is an important safety improvement.

Many factors must be taken into account when performing a safe procedure. Although each endoscopy has its own precautions and preparations, many are common and can be divided into the following three major procedural stages: pre-procedure, intra-procedure and post-procedure. While initiating our study, during the baseline phase, it became obvious that adding a checklist to the endoscopy unit’s current practices would result in low compliance, hence, intervention methods were required for reaching the goal of a minimum compliance of 75%. As the study continued, we noticed that immediate and targeted feedback seems to be a useful tool for dealing with low compliance. Team feedback in surgery was shown to improve technical performance and reduce adverse events. Post-procedural team feedback is useful, especially in the case of a significant adverse event. In our study, the feedback offered by all team members revealed that the improvement of the checklist implementation is credited to the designated individual assuming ownership for the completion and being accountable for involving the entire multidisciplinary team at each “time out” and “sign in” checklist completion. Even though the results of the staff questionnaire were not statistically significant due to the small number of participants, both physicians and nurses offered consistent and valuable responses, all supporting the usefulness of the endoscopy safety checklist.

The following two main variables of interest were identified in our study: colonic preparation–colonoscopy completion rate and colonic preparation–adenoma detection rate. Both the colonoscopy completion rate and the adenoma detection rate are positively influenced by a proper colonic preparation (Boston Bowel Preparation Scale 2 or 3 for each colonic segment) and, on the other hand, a poor colonic preparation (Boston Bowel Preparation Scale 0 or 1 for each colonic segment) will lead to a poor adenoma detection rate and/or an incomplete colonoscopy evaluation.

Due to a relatively short study period, the compact and constant number of the subjects involved in completing the safety checklist and the staff questionnaire associated with the clear protocol regarding the data collection, we did not register any missing data, which might represent one of the study strengths. A limitation of our study might be the sample size, as the number of GI endoscopy procedures were restricted by the COVID-19 pandemic. An ongoing audit is necessary, ideally with a bigger sample size, to enable subgroup analysis and reveal particular weak points or problematic practices. The baseline measurements included 126 procedures, while post-intervention, 446 procedures were assessed. To strengthen the validity of the findings, it would be advantageous to collect comparable numbers at the study’s baseline and post-intervention periods if it were to be conducted again. The “Hawthorne effect,” which occurs when team members become aware that they are being watched, is an observation bias and another limitation of the study’s scope, as the outcomes might not accurately reflect the everyday surveillance-free practice in the endoscopy lab. The installation of cameras in the endoscopy rooms and using remote video auditing, like in the study of Raphael et al. [22], can help to fight the observation bias. Regarding the survey conducted for assessing the team satisfaction and perception of the checklist, we could think about recall bias, as the questionnaire was handed out to the staff members at the end period of the study. In this case, the memory of the initial experiences with the checklist might be diluted or underestimated versus the more recent and positive experiences after adapting to the checklist requirements. In order to address the potential confirmation bias, the authors of this paper were not included in the observational study as subjects of the GI endoscopy team.

Although the study achieved its objectives, there is still an opportunity for further development. This ought to enable more focused initiatives to lessen the standard-setting variation.

In the end, we acknowledge that a customized approach and patient involvement was necessary for understanding the entire patient experience throughout the GI endoscopy department and how the endoscopy unit performed. Although there were still some items on the checklist that needed to be completed, overall, the compliance rates among the team members increased during the study period, which is very encouraging.

## 5. Conclusions

Using a checklist and adapting it to local conditions is a high-level recommendation of the Romanian Ministry of Health. In a medical world where safety and quality are essential, a checklist could prevent medical errors, and team time out can ensure high-quality endoscopies, enhance teamwork and offer patients confidence in the medical team. The checklist implementation, in our labs, significantly strengthened the team, flattened hierarchies and improved patient approach and team communication. In a medical world where safety and quality are paramount, a checklist, implemented in all endoscopy services, could prevent procedural errors, streamline teamwork, ensure high-quality work and provide confidence to patients. Hence, standardized methods, such as checklists, promoting patient safety culture and encouraging patient involvement, are strategies for augmented patient safety.

## Figures and Tables

**Table 1:** Facilitators and barriers to the implementation of endoscopy safety checklist.

Facilitators	Barriers
✔ Concise and specific safety checklist ✔ Rising awareness among the entire team regarding quality control and safety in endoscopy ✔ Senior physician/professor leadership ✔ Endoscopic procedures performed during the first part of the working day ✔ Daily nomination of a team member responsible for checklist ✔ The young age of the person nominated to be responsible for the checklist ✔ Minimize distractions in the endoscopy room ✔ Immediate targeted feedback ✔ Constant checklist reassessment and fast adjustments in the daily practice	✔ Irrelevant safety checklist items ✔ The additional time required to complete the checklist ✔ Fear that additional time spent per procedure will lead to less funding ✔ Deficient patient safety culture ✔ Team resistance to change ✔ Loss of physician autonomy ✔ Lack of leadership ✔ Lack of a designated member responsible for the checklist ✔ Fear of accentuating patient anxiety

**Table 2:** Study outcome rates, *n* = numeric value; 126 baseline procedures, 446 post-intervention procedures.

Study Outcomes	Baseline Rates (Yes %)	Yes *n*/126	No *n*/126	Post-Intervention Rates (Yes%)	Yes *n*/446	No *n*/446	*p*-Value
Checklist completion rate	24.60	31	95	89.01	397	49	The chi-square statistic is 216.37. The *p*-value is < 0.01. The result is significant at *p* < 0.05.
Patient’s identity verification (by nurse)	97.62	123	3	98.88	441	5	The chi-square statistic is 1.1308. The *p*-value is 0.28. The result is not significant at *p* < 0.05.
Patient’s identity verification (by endoscopist)	15.87	20	106	90.13	402	44	The chi-square statistic is 280.04. The *p*-value is <0.01. The result is significant at *p* < 0.05.
Validity of the procedure appropriateness	87.30	110	16	93.95	419	27	The chi-square statistic is 6.23. The *p*-value is 0.012. The result is significant at *p* < 0.05.
Documented proper colonic preparation	76.98	97	29	90.81	405	41	The chi-square statistic is 17.47. The *p*-value is <0.01. The result is significant at *p* < 0.05.
Colonoscopy completion rate	97.62	123	3	98.88	441	5	The chi-square statistic is 17.47. The *p*-value is <0.01. The result is significant at *p* < 0.05.
Peri-procedural complications	0.79	1	125	0.45	2	444	The chi-square statistic is 0.22. The *p*-value is 0.63. The result is not significant at *p* < 0.05.
Adequate histological labeling management	48.67	110	16	93.72	418	28	The chi-square statistic is 180.79. The *p*-value is <0.01. The result is significant at *p* < 0.05.
Explicit recording of follow-up recommendations	69.84	88	38	95.07	424	22	The chi-square statistic is 66.58. The *p*-value is <0.01. The result is significant at *p* < 0.05.

**Table 3:** Staff perception regarding the checklist implementation (physicians n = 15, nurses n = 8).

	Yes *n* (%)		No *n* (%)		Hesitant *n* (%)		*p*-Value
	Physicians	Nurses	Physicians	Nurses	Physicians	Nurses	
In your opinion, did the checklist cause a time delay?	2 (13.3%)	3 (37.5%)	10 (66.6%)	5 (62.5%)	3 (20%)	0	The chi-square statistic is 1.11. The *p*-value is 0.29. The result is not significant at *p* < 0.05.
In your opinion, did the checklist improve team communication and teamwork?	11 (73.3%)	7 (87.5%)	1 (6.6%)	0	3 (20%)	1 (12.5%)	The Fisher’s exact test statistic value is 1. The result is not significant at *p* < 0.05.
In your opinion, did the checklist improve patient safety?	13 (86.66%)	7 (87.5%)	1 (6.66%)	0	1 (6.66%)	1 (12.5%)	The Fisher’s exact test statistic value is 1. The result is not significant at *p* < 0.05.
Would you consider the checklist useful if you were to have an endoscopy?	14 (93.3%)	6 (75%)	0	1 (12.5%)	1 (6.6%)	1 (12.5%)	The Fisher’s exact test statistic value is 0.33. The result is not significant at *p* < 0.05.
Would you declare yourself satisfied with the checklist implementation process?	10 (66.6%)	5 (62.5%)	3 (20%)	0	2 (13.3%)	3 (37.5%)	The Fisher’s exact test statistic value is 0.52. The result is not significant at *p* < 0.05.

## Data Availability

All data supporting the study can be located in the archive of the Research Center of Gastroenterology and Hepatology Craiova, Romania and the Lotus Image Medical Center, Actamedica SRL, Targu Mures, Romania.
